# Supplementary material for: Bridging scales in a multiscale pattern-forming system
Source: Proc Natl Acad Sci U S A. 2022 Aug 12;119(33):e2206888119. doi: 10.1073/pnas.2206888119 (PMC9388104; doi:10.1073/pnas.2206888119)
Supplement: Supplementary File [file pnas.2206888119.sapp.pdf]

PNAS
www.pnas.org

Supplementary Information for

Bridging scales in a multiscale pattern-forming system

Laeschkir Würthner*, Fridtjof Brauns*, Grzegorz Pawlik, Jacob Halatek, Jacob Kerssemakers, Cees Dekker, Erwin Frey

Corresponding authors:
Erwin Frey
E-mail: frey@lmu.de,
Cees Dekker
E-mail: c.dekker@tudelft.nl

***these authors contributed equally to this work.**

This PDF file includes:

Supplementary text
Figs. S1 to S4
Legends for Movies S1 to S9
SI References

Other supplementary materials for this manuscript include the following:

Movies S1 to S9

Supporting Information Text

Linear stability analysis

As outlined in the main text, we performed a linear stability analysis to determine the instantaneous dispersion relation in each slice of the wedge geometry. The dispersion relation, from which we determine the commensurability of unstable modes, then informs about the pattern type in the slice. The general procedure for the linear stability analysis is as follows: First, one determines a homogeneous steady state and linearizes the dynamics around this steady state. The linearized system can be solved by a *normal mode expansion*, which yields a relation between the growth rate of modes σ and the mode number q. The relationship $\sigma(q)$ is the dispersion relation, and indicates whether spatial perturbations with mode number q decay to the homogeneous steady state ($\sigma < 0$) or are amplified and growth exponentially with time ($\sigma > 0$). In the following, we briefly explain the linear stability analysis procedure for the Min dynamics in rectangular geometry and refer for an in-depth analysis to Refs. (1, 2)

Homogeneous steady states at the membrane. To obtain the homogeneous steady state solution along the membrane, we first determine the the steady state concentration profiles in the cytosol $\mathbf{c}^* = [c^*_{\mathrm{D}}, c^*_{\mathrm{DD}}, c^*_{\mathrm{E}}]^\top$:

$$\mathbf{0} = D_c(\partial_x^2 + \partial_z^2)\mathbf{c}^* - \beta\mathbf{c}^*, \qquad \text{[S1]}$$

where $\beta = \mathrm{diag}(0, \lambda, 0)$. These equations can be solved by a separation of variables and yield the following solution:

$$c^*_{\mathrm{D}}(z) = c^*_{\mathrm{D}}|_{z=0} = \text{const.}, \qquad \text{[S2a]}$$

$$c^*_{\mathrm{DD}}(z) = c^*_{\mathrm{DD}}|_{z=0}\frac{\cosh\left((H-z)/\ell\right)}{\cosh\left(H/\ell\right)}, \qquad \text{[S2b]}$$

$$c^*_{\mathrm{E}}(z) = c^*_{\mathrm{E}}|_{z=0} = \text{const.}, \qquad \text{[S2c]}$$

where $c^*|_{z=0} = c^*_i(z=0)$ ($i \in \{\mathrm{D}, \mathrm{DD}, \mathrm{E}\}$) denote the steady state homogeneous cytosolic concentrations at the membrane, and $\ell = \sqrt{D_c/\lambda}$ defines the characteristic length scale of cytosolic gradients into the bulk. Note that $\partial_x \mathbf{c}^* = 0$, because we are interested in homogeneous solutions along the membrane. Plugging these solutions into the membrane reactions and bulk-membrane coupling (cf. Eq. [2] and [3] in the main text) one finds the following set of equations:

$$\boldsymbol{f}\left(\mathbf{c}^*|_{z=0}, \mathbf{m}^*\right) = \boldsymbol{\Phi}, \qquad \text{[S3a]}$$

$$\mathbf{r}(\mathbf{c}^*|_{z=0}, \mathbf{m}) = \mathbf{0}, \qquad \text{[S3b]}$$

where

$$\boldsymbol{\Phi} = \begin{bmatrix} 0 \\ D_c/\ell \tanh\left(H/\ell\right) c^*_{\mathrm{DD}}|_{z=0} \\ 0 \end{bmatrix}. \qquad \text{[S4]}$$

Together with the mass-conservation constraint:

$$\overline{n}_{\mathrm{D}} = c^*_{\mathrm{D}}|_{z=0} + \frac{1}{H}(m^*_{\mathrm{d}} + m^*_{\mathrm{de}}), \qquad \text{[S5a]}$$

$$\overline{n}_{\mathrm{E}} = c^*_{\mathrm{E}}|_{z=0} + \frac{1}{H}m^*_{\mathrm{de}}, \qquad \text{[S5b]}$$

the solution of the nonlinear system Eq. [S3] and Eq. [S5] determines the homogeneous steady state solutions at the membrane. This nonlinear system of equations can be solved numerically by root-finding algorithms such as the *Newton algorithm*. Here, we used the built-in function `NSolve[]` in Mathematica 12 to numerically determine the homogeneous steady states.

The linearized dynamics and growth rates for small spatial perturbations. Next, we ask for the stability of the homogeneous steady state against small spatial perturbations. To this end, we linearize the dynamics around the homogeneous steady state and determine the time evolution of perturbations $\delta\mathbf{m}(x,t) = \mathbf{m}(x,t) - \mathbf{m}^*$ and $\delta\mathbf{c}(x,z,t) = \mathbf{c}(x,z,t) - \mathbf{c}^*|_{z=0}$:

$$\partial_t \delta\mathbf{c} = D_c(\partial_x^2 + \partial_z^2)\delta\mathbf{c} - \beta\delta\mathbf{c}, \tag{S6a}$$

$$-D_c\partial_z\delta\mathbf{c}\big|_{z=0} = \partial_{\mathbf{u}}\mathbf{f}\,\delta\mathbf{u}, \tag{S6b}$$

$$\partial_t\delta\mathbf{m} = D_m\partial_x^2\delta\mathbf{m} + \partial_{\mathbf{u}}\mathbf{r}\delta\mathbf{u}, \tag{S6c}$$

where $\mathbf{u} = [\mathbf{c},\mathbf{m}]^\top$ denotes the concentration vector and $\delta\mathbf{u} = [\delta\mathbf{c}|_{z=0}, \delta\mathbf{m}]^\top$ the perturbation vector at the membrane. The Jacobian matrices of the bulk-boundary coupling and membrane reactions $\partial_{\mathbf{u}}\mathbf{f} \equiv \partial_{\mathbf{u}}\mathbf{f}|_{(\mathbf{c}^*|_{z=0},\mathbf{m}^*)}$ and $\partial_{\mathbf{u}}\mathbf{r} \equiv \partial_{\mathbf{u}}\mathbf{r}|_{(\mathbf{c}^*|_{z=0},\mathbf{m}^*)}$ are evaluated at the homogeneous steady state at the membrane. The linearized system Eq. [S6] can be solved by a normal mode expansion of the form:

$$\delta c_i(x,z,t) = \sum_q e^{\sigma_q t}\cos(qx) Z_i(z;\sigma_q,q)\delta\hat{c}_{i,q}, \tag{S7a}$$

$$\delta\mathbf{m}(x,t) = \sum_q e^{\sigma_q t}\cos(qx)\delta\hat{\mathbf{m}}_q,, \tag{S7b}$$

here σ_q defines the growth rate of perturbations with respective mode number q. The Fourier coefficients are given by $\delta\hat{\mathbf{c}}_q$ and $\delta\hat{\mathbf{m}}_q$, respectively, and the bulk modes $Z_i(z;\sigma_q,q)$ have a similiar form as for the homogeneous steady states:

$$Z_i(z;\sigma_q,q) \sim \frac{\cosh(\gamma_q^i(z-H))}{\cosh(\gamma_q^i H)}, \tag{S8}$$

where the parameters γ_q^i take different values dependent on whether the bulk dynamics is purely diffusive or contains linear reactions (i.e. nucleotide exchange), therefore $\gamma_q^{\mathrm{D}} = \gamma_q^{\mathrm{E}} = \sqrt{\sigma_q/D_c + q^2}$ and $\gamma_q^{\mathrm{DD}} = \sqrt{(\sigma_q+\lambda)/D_c + q^2}$, respectively. Plugging the normal mode expansions Eq. [S7] into the linearized bulk-boundary coupling and membrane dynamics Eq. [S6] yields an eigenvalue problem for the growth rates σ_q as a function of the modes q. This transcendental eigenvalue problem is given in compact form by:

$$\underbrace{\begin{pmatrix} -D_c\,\mathbf{\Gamma}(\sigma_q,q) + \mathbf{f_c} & \mathbf{f_m} \\ \mathbf{r_c} & -(\sigma_q + q^2 D_m)I_2 + \mathbf{r_m}\end{pmatrix}}_{=:\,\mathbf{M}(\sigma_q,q)} \begin{bmatrix}\delta\hat{\mathbf{c}}_q \\ \delta\hat{\mathbf{m}}_q\end{bmatrix} = \mathbf{0} \tag{S9}$$

where I_2 denotes the 2x2 identity matrix, and $\mathbf{\Gamma} := \mathrm{diag}\left(\Gamma(\gamma_q^{\mathrm{D}}), \Gamma(\gamma_q^{\mathrm{DD}}), \Gamma(\gamma_q^{\mathrm{E}})\right)$ is a coupling matrix due to bulk-coupling, where

$$\Gamma(\gamma_q^i) = D_c\gamma_q^i\tanh\left(\gamma_q^i H\right). \tag{S10}$$

The growth rates σ_q are then determined by solving the transcendental characteristic equation:

$$\det\left(\mathbf{M}(\sigma_q,q)\right) = 0. \tag{S11}$$

The dispersion relation is given by the largest real solution (fastest growing mode) of Eq. [S11]. We solved Eq. [S11] numerically using the built-in function `FindRoot[]` in Mathematica 12.

Pattern prediction from local dispersion relations

Here, we provide more details on how we technically predict the patterns in each slice from the simulation data. Specifically, we explain the steps required to generate the kymographs in the main text (Figs. 4 and 5)

Computation of the total densities in the slices from numerical data. To determine the slice averaged densities at each position x along the wedge geometry, we first exported the membrane concentration profiles of MinD and MinE from the simulation data as a *grid data file*. We exported the grid file for each time step $\delta t = 2\,\mathrm{s}$, where the grid file consists of 400×400 spatial points (400 data points in each spatial direction), which is equivalent to a spatial discretization of $\delta x = \delta y = 1.25\,\mu\mathrm{m}$.

To obtain the slice-averaged densities, we imported the simulation data to *Mathematica 12* and averaged the total densities of MinD and MinE along the $y-$direction, thereby reducing the data to a one-column grid file which contains 400 points, corresponding to the slice-averaged densities along the $x-$direction. To reduce the computational effort for the calculations of the dispersion relations and to smooth the data, we additionally averaged the slice densities over 4 points in space (i.e. resulting in a new step size $\delta x_{\mathrm{avg}} = 4\delta x$) and 10 steps in time (i.e. obtaining the new time step $\delta t_{\mathrm{avg}} = 10\delta t$) using the Mathematica built-in functions `Partition[]` and `Mean[]`. This way we reduced the number of points in the grid file to 100 spatial points and 450 points in time, respectively.

Determination of the instantaneous dispersion relations and commensurability condition. The total average densities in each slice (see above) are then plugged into Eq. [S11] to determine the dispersion relation at each point in time. From the disperion relation we determine the commensurability of unstable modes, whose value informs about the pattern in the slices as explained in the main text. To illustrate how the commensurability condition varies with time and space, we generated the kymographs (Figs. 4A and 5C) in the main text, where the commensurability condition is color coded as shown in the figure.

Since the transition in the commensurability condition from standing wave to traveling wave patterns is not sharp, but rather occurs around the onset for $q_{\mathrm{max}}/q^* = 2$, we additionally applied a color gradient for standing waves (green) to make this point clear.

Regularization of the local-equilibrium approximation. In the main text, we use a local-equilibrium approximation to obtain the reduced dynamics for the (slice-averaged) total densities. This reduced dynamics is well-defined as long as the local equilibria c_i^* do not undergo saddle-node bifurcations (where a stable and an unstable equilibrium annihilate). If a saddle-node bifurcation occurs at some point in the spatial domain, $c_i^*(x)$ will be discontinuous at this point as it jumps from one branch of equilibria (which is annihilated) to another one. To regularize the dynamics, we introduce auxiliary fields $\tilde{c}_i(x,t)$ which relax to the equilibrium concentrations $c_i^*(x,t)$ on a fast timescale compared to the large scale mass-redistribution. In the limit of fast relaxation, the concrete implementation of the relaxation dynamics is irrelevant. An intuitive choice for the relaxation dynamics would be, for example, $\partial_t \tilde{c}_i(x,t) = -\alpha[\tilde{c}_i(x,t) - c_i^*(x,t)]$, where α denotes the relaxation rate. However, this choice requires explicit computation of the local equilibria $c_i^*(x,t)$ at each timestep which is computationally costly and complicates the numerical implementation.

In the following, we construct an auxiliary relaxation dynamics such that the equilibria c_i^* are implicit in the relaxation dynamics and don't need to be computed explicitly. We start by imposing the local equilibrium assumption on the vertical bulk profiles (cf. Eq. [S2]). Note that we do this at each lateral position x separately. For ease of notation, we do not denote the

x-dependence explicitly below.

$$\tilde{c}_{\mathrm{D}}(z) = \tilde{c}_{\mathrm{D}} = \text{const.} \tag{S12a}$$

$$\tilde{c}_{\mathrm{DD}}(z) = \tilde{c}_{\mathrm{DD}}(0)\, \frac{\cosh\Big(\sqrt{\lambda/D_c}\,(H(x)-z)\Big)}{\cosh\Big(\sqrt{\lambda/D_c}\,H(x)\Big)}\,, \tag{S12b}$$

$$\tilde{c}_{\mathrm{E}}(z) = \tilde{c}_{\mathrm{E}} = \text{const.} \tag{S12c}$$

The dynamics for the total densities is now defined in terms of the auxiliary cytosolic densities (cf. Eq. [8] in the main text)

$$\partial_t \tilde{n}_{\mathrm{i}}(x,t) = D_c \partial_x^2 \tilde{c}_{\mathrm{i}} + D_c \tfrac{\partial_x H(x)}{H(x)}\, \partial_x \tilde{c}_{\mathrm{i}}\,. \tag{S13}$$

To construct the auxiliary relaxation dynamics for $\tilde{c}_{\mathrm{D}}$ and $\tilde{c}_{\mathrm{E}}$, we eliminate the membrane concentration variables m_{d}, m_{de} by using the mass conservation constraint (cf. 4a and 4b in the main text)

$$\tilde{m}_{\mathrm{d}} = H(x)\,\tilde{n}_{\mathrm{D}} - H(x)\,\tilde{c}_{\mathrm{D}} - \tilde{m}_{\mathrm{de}}, \tag{S14}$$

$$\tilde{m}_{\mathrm{de}} = H(x)\,\tilde{n}_{\mathrm{E}} - H(x)\,\tilde{c}_{\mathrm{E}}, \tag{S15}$$

Next, we eliminate $\tilde{c}_{\mathrm{DD}}(0)$ by imposing the boundary condition $\partial_z \tilde{c}_{\mathrm{DD}}|_{z=0} = k_{\mathrm{de}} m_{\mathrm{de}}$, which yields

$$\sqrt{\lambda/D_c}\,\tanh\Big(\sqrt{\lambda/D_c}\,H(x)\Big)\,\tilde{c}_{\mathrm{DD}}(0) = k_{\mathrm{de}}\tilde{m}_{\mathrm{de}}. \tag{S16}$$

Note that we do not enforce the boundary conditions for $\tilde{c}_{\mathrm{D}}$ and $\tilde{c}_{\mathrm{E}}$. Instead, we will use these boundary conditions to define auxiliary relaxation dynamics for $\tilde{c}_{\mathrm{D}}$ and $\tilde{c}_{\mathrm{E}}$ as follows

$$\partial_t \tilde{c}_{\mathrm{i}}(x,t) = D_c \partial_x^2 \tilde{c}_{\mathrm{i}} + D_c \tfrac{\partial_x H(x)}{H(x)}\, \partial_x \tilde{c}_{\mathrm{i}} + \alpha \tilde{f}_{\mathrm{i}}\big(\tilde{c}_{\mathrm{D}}, \tilde{c}_{\mathrm{E}}\big)\,, \tag{S17}$$

with the auxiliary reaction terms

$$\tilde{f}_{\mathrm{D}} = \frac{1}{H}\big(k_{\mathrm{de}}\tilde{m}_{\mathrm{de}} - (k_{\mathrm{D}} + k_{\mathrm{dD}}\tilde{m}_{\mathrm{d}})[\tilde{c}_{\mathrm{D}} - \tilde{c}_{\mathrm{DD}}(0)]\big), \tag{S18}$$

$$\tilde{f}_{\mathrm{E}} = \frac{1}{H}\big(k_{\mathrm{de}}\tilde{m}_{\mathrm{de}} - k_{\mathrm{dE}}\tilde{m}_{\mathrm{d}}\tilde{c}_{\mathrm{E}}\big) \tag{S19}$$

obtained by substituting the auxiliary variables into the boundary fluxes of c_i (see Eq. [11] in the main text). Observe that $\tilde{f}_{\mathrm{i}} = 0$ for $\tilde{c}_{\mathrm{i}} = c_{\mathrm{i}}^*$, i.e. the auxiliary reactions relax towards the local steady state concentrations, as required. The factor $1/H$ comes in because the auxiliary fields $\tilde{c}_i$ represent the uniform bulk concentrations whose rate of change is obtained by distributing the boundary flux f_i over the entire vertical column with height H. The relaxation rate factor α above can be used to adjust the relaxation rate to minimize the deviation from the local equilibria while avoiding the emergence of too sharp gradients. We performed simulations of the auxiliary dynamics for different values of α and found no noticeable changes in the results, when increasing α above 1.

We have numerically implemented the system of PDEs defined by Eq. [S13] and Eq. [S17] in *Mathematica 12.3* using a finite-difference discretization (first order central differences, 200 grid points). The resulting high-dimensional ODEs system is integrated using *Mathematica's* `NDSolve` function.

Average total densities control the final steady state pattern

In the main text, we have shown that a variety of different patterns emerge on the membrane, and that these patterns transition to other patterns over time. For large times, however, we found that the system approaches a stable steady state that is characterized by standing wave patterns which emerge on the entire membrane. The underlying reason is that for large times the density profile in the bulk approaches a (heterogeneous) steady state distribution due to mass-redistribution (diffusive fluxes). At this steady state, the dispersion relation becomes insensitive to the local total densities and the bulk height, resulting in loss of heterogeneity and thus to the selection of one pattern type on the membrane. What determines the type of this pattern?

The final steady state profiles of the total densities depend on the average total densities in the system, which are set by the initial condition. For the simulation presented in the main text, we tuned these densities to achieve $q_{\mathrm{max}} \approx 2q^*$ (i.e. standing waves) in the final steady state. As the density profiles relax towards this steady state, they fluctuate (oscillate) around this critical mode ratio which leads to the intriguing sequence of transient patterns.

For comparison, we performed a second simulation with a lower average MinD density $\bar{n}_{\mathrm{D}} = 638\,\mathrm{\mu m}^{-3}$, such that the $q_{\mathrm{max}} > 2q^*$ in the final steady state. Accordingly, the system settles in traveling wave patterns after a considerably shorter transient (see Fig. S1 and Movie S1).

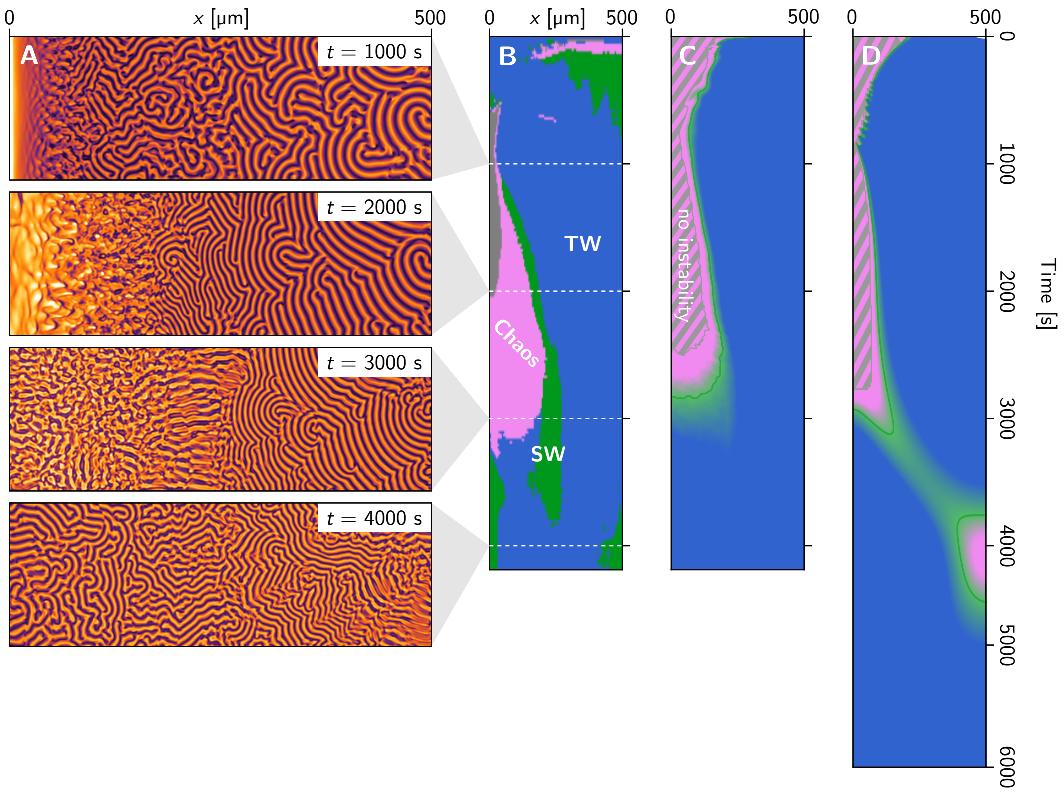

Fig. S1. Pattern type classification and prediction for a second parameter set which exhibits traveling waves in the entire domain in the steady state reached for large times ($t > 4000\,\mathrm{s}$). (A) Snapshots from the full numerical simulation (cf. Movie S1). Parameters: $\bar{n}_\mathrm{D} = 638\,\mu\mathrm{m}^{-3}$, all other parameters as in Tab. 1 in the main text. (B) Computer-based pattern classification from the full simulation using *ilastik* (compare to Fig. 4D). Note that the classifier is unreliable during the initial transient (first ∼500 s) where large scale trigger waves dominate. (C) Prediction based on slice-averaged total densities extracted from the full numerical simulation (analogous to Fig. 4A). (D) Prediction based on the reduced mass-redistribution dynamics Eq. [8] (analogous to Fig. 5C).

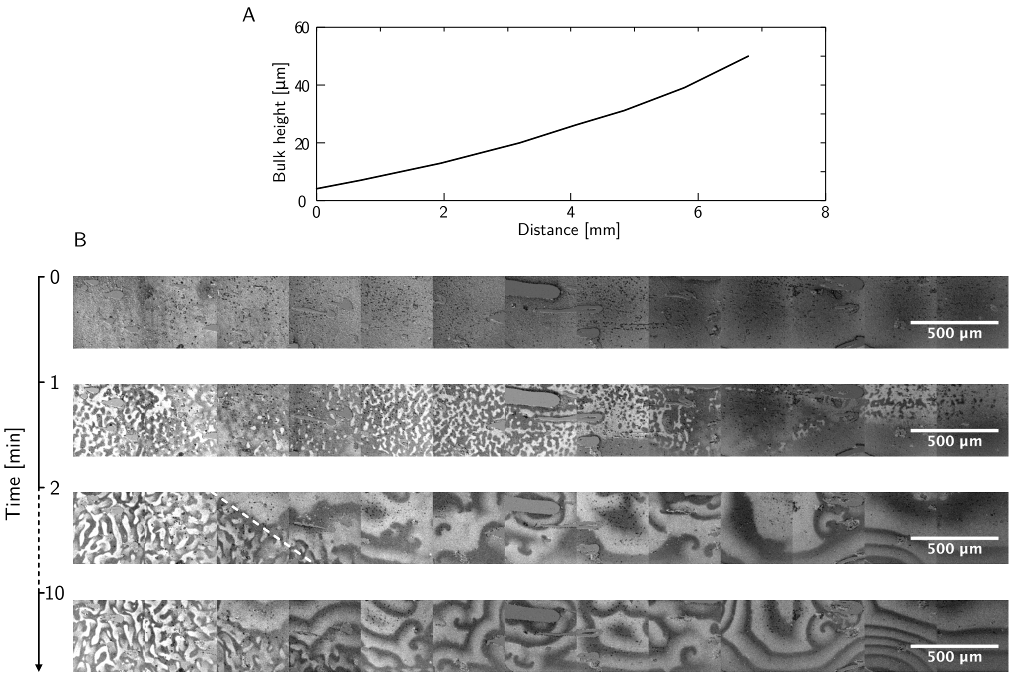

Fig. S2. Establishment of coexisting patterns in different spatial regions. (*A*) Measured bulk height profile versus lateral distance. (*B*) Spatial intensity profile of MinD along the wedge at different points in time, snapshots were taken at 0, 1, 2, and 10 minutes. White dashed line shows the approximate boundary line, where standing wave patterns transition to homogeneous oscillations.

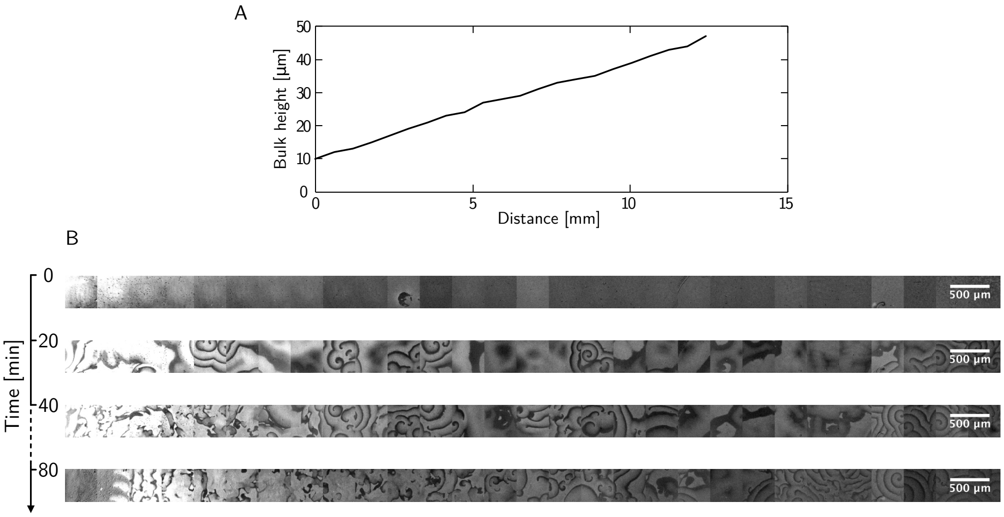

Fig. S3. Transition of homogeneous oscillations to traveling wave patterns. (*A*) Measured bulk height profile versus lateral distance. Snapshots along the wedge were taken at the time 0, 20, 40 and 80 minutes. (*B*) Spatial intensity profile of MinD along the wedge at different points in time, snapshots were taken at 0, 20, 40, and 80 minutes. At early times, homogenous oscillations turn into travelling waves at different regions. For long times, regions containing rather chaotic homogenous oscillations invade other regions (that contain different patterns) from low to high bulk heights.

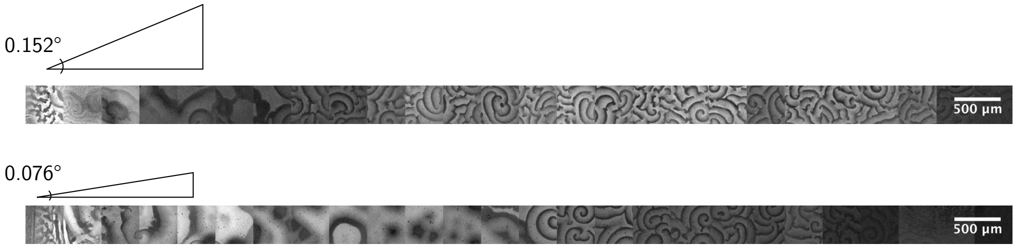

Fig. S4. The bulk height gradient affects patterns along the wedge. Shown are the Min patterns in two different flow cells that had a different tilt (angle between top and bottom membranes). In the setup with higher tilt (top), traveling wave patterns are more abundant than homogenous oscillations. For a smaller angle (bottom), one observes more regions that contain homogeneous oscillations. The snapshots were both taken 20 minutes after flushing in the Min proteins (MinD channel shown).

Movie S1. Numerical simulation of the Min dynamics in wedge geometry for total average densities $\bar{n}_{\mathrm{D}} = 665\,\mu\mathrm{m}^{-3}$ and $\bar{n}_{\mathrm{E}} = 410\,\mu\mathrm{m}^{-3}$. Shown is the MinD density along the membrane (bottom surface of the wedge, see Fig. 1B). For large times, the system approaches a steady state consisting of standing wave patterns on the entire membrane surface.

Movie S2. Numerical simulation for total average densities $\bar{n}_{\mathrm{D}} = 638\,\mu\mathrm{m}^{-3}$, $\bar{n}_{\mathrm{E}} = 410\,\mu\mathrm{m}^{-3}$. For these parameters, traveling wave patterns form in the entire wedge in the steady state reached for large times ($t > 4000\,\mathrm{s}$). Compare Fig. S1.

Movie S3. Experimentally observed Min patterns in a wedge-shaped microfluidic flow chamber. As in our numerical simulations, we observe coexisting spatiotemporal patterns along the membrane (as shown in Fig. 2C).

Movie S4. Experimentally observed establishment of a sharp boundary between regions containing traveling wave patterns and regions containing chaotic or standing wave patterns (as shown in Fig. S2).

Movie S5. Experiment showing emergence of homogeneous oscillations and transitions to traveling waves (corresponds to Fig. S3).

Movie S6. Experiments with steep bulk height gradients show the predominant emergence of traveling wave patterns (cf. Fig. S4, top).

Movie S7. Experiments with shallow bulk height gradients show more regions with nearly homogeneous oscillations/phase waves (cf. Fig. S4, bottom).

Movie S8. Pattern prediction from regional dispersion relations and coarse-grained densities (as illustrated in Fig. 3 and Fig. 4).

Movie S9. Numerical simulation of the Aranson–Tsimring model showing the order parameter amplitude $|\psi|$ (top) and coarse grained density (bottom). Dashed white line indicates the stability threshold determined from regional dispersion relations. See Fig. 6 for details.

References

1. J Halatek, E Frey, Rethinking pattern formation in reaction–diffusion systems. *Nat. Phys.* **14**, 507 (2018).
2. F Brauns, et al., Bulk-surface coupling identifies the mechanistic connection between min-protein patterns in vivo and in vitro. *Nat. Commun.* **12** (2021).
